# DNA damage accumulation and repair defects in FLT3‐ITD acute myeloid leukemia: Implications for clonal evolution and disease progression

**DOI:** 10.1002/hon.3076

**Published:** 2022-09-28

**Authors:** Francisco Alejandro Lagunas‐Rangel

**Affiliations:** ^1^ Department of Surgical Sciences Uppsala University Uppsala Sweden

**Keywords:** AML, FLT3 inhibitor, homologous recombination, NHEJ, poor clinical prognosis, ROS

## Abstract

Acute myeloid leukemia is a group of hematological diseases that have a high mortality rate. During the development of this pathology, hematopoietic cells acquire chromosomal rearrangements and multiple genetic mutations, including FLT3‐ITD. FLT3‐ITD is a marker associated with a poor clinical prognosis and involves the activation of pathways such as PI3K/AKT, MAPK/ERK, and JAK/STAT that favor the survival and proliferation of leukemic cells. In addition, FLT3‐ITD leads to overproduction of reactive oxygen species and defective DNA damage repair, both implicated in the appearance of new mutations and leukemic clones. Thus, the purpose of this review is to illustrate the molecular mechanisms through which FLT3‐ITD generates genetic instability and how it facilitates clonal evolution with the generation of more resistant and aggressive cells. Likewise, this article discusses the feasibility of combined therapies with FLT3 inhibitors and inhibitors of DNA repair pathways.

## 1 INTRODUCTION

Acute myeloid leukemia (AML) is a group of hematologic diseases that result from clonal transformation of hematopoietic precursors through the acquisition of chromosomal rearrangements and multiple genetic mutations that confer proliferative and survival advantages along with a negative effect on differentiation.^1^, ^2^, ^3^ It is estimated that approximately 28% of all leukemias worldwide correspond to AML, being the predominant form of leukemia in the neonatal and adult periods, but representing a small fraction of cases during childhood and adolescence.^4^ Remarkably, AML presents historically high mortality rates that, despite advances in treatment, have not managed to increase patient survival as expected.^5^ Features associated with a poor prognosis include the acquisition of certain chromosomal rearrangements such as t(6; 9) (p23; q34.1);*DEK‐NUP214*, inv(3) (q21.3q26.2); *GATA2, MECOM* and t(1; 22) (p13.3; q13.3);*RBM15‐MKL1*, as well as mutations in genes such as *FLT3*.^6^

**FIGURE 1:**
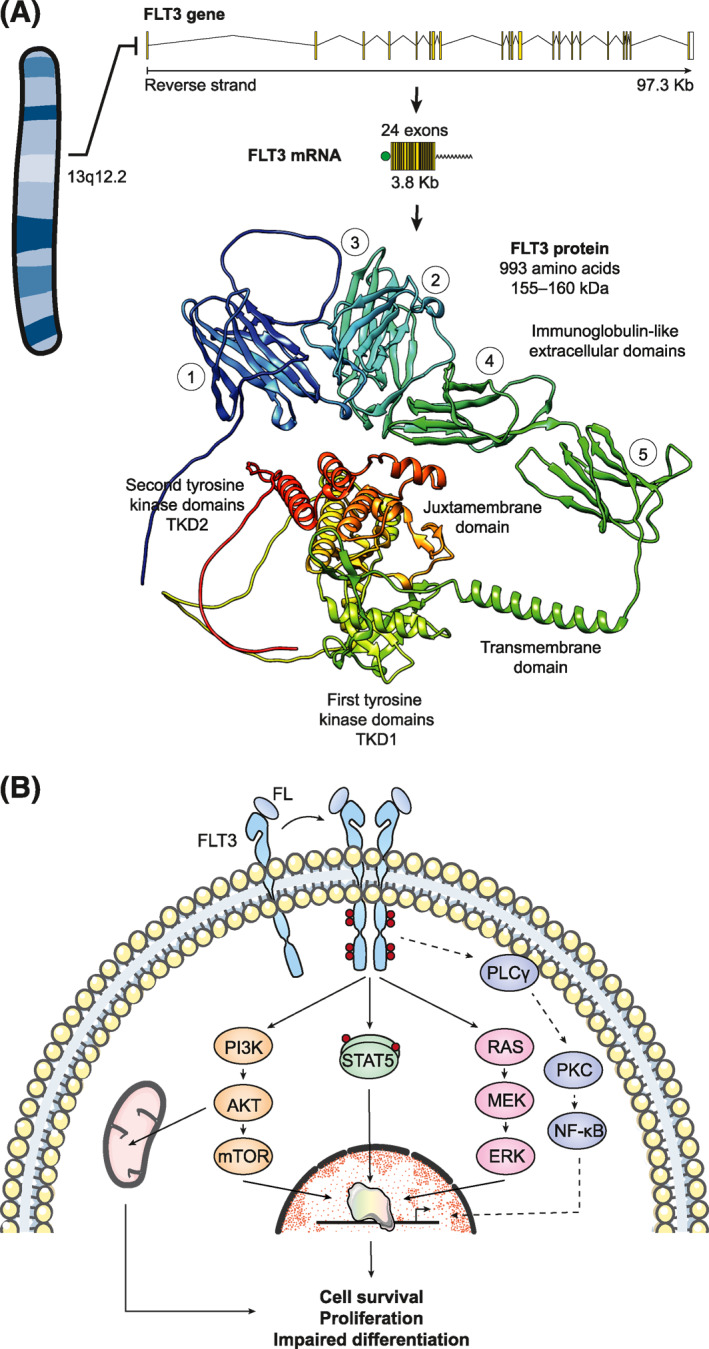
Structure and signaling of FLT3. (A), The FLT3 gene is located on chromosome 13 and extends for 97.3 Kb. It contains 24 exons that are transcribed into a mature mRNA of 3.8 Kb and encode a protein of 993 amino acids. The FLT3 protein is a receptor tyrosine kinase that has five extracellular immunoglobulin‐like domains, a transmembrane domain, a juxtamembrane domain, and two tyrosine kinase domains separated by an insert kinase domain. (B), FLT3 signaling involves its binding to FL, causing conformational changes leading to a catalytically active protein. FLT3 activates multiple pathways such as PI3K/AKT, MAPK/RK, JAK/STAT, and PLC that provide signals for survival, proliferation, and altered differentiation

FMS‐like tyrosine kinase 3 (FLT3) is a membrane‐bound receptor tyrosine kinase (RTK) that belongs to the RTK subclass III family along with KIT, CSF1R, PDGFRA, and PDGFRB.^7^ This protein is mainly expressed in lymphohematopoietic organs such as bone marrow, thymus, lymph nodes, liver, spleen, and placenta and is involved in important processes of hematopoietic cells, such as proliferation, differentiation, and survival.^2^ Notably, FLT3 expression in normal human cells occurs primarily in early myeloid and lymphoid progenitors and disappears in differentiated cells.^8^ Structurally, FLT3 exhibits five extracellular immunoglobulins‐like domains, a single‐pass transmembrane domain, a juxtamembrane domain (JM), and two intracellular tyrosine kinase domains (TKDs) linked by an insertion kinase domain (Figure 1A).^9^ FLT3 signaling involves the binding of this receptor to its ligand (FL) through contacts between the third immunoglobulin‐like domain of FLT3 and the N‐terminal segment of FL.^10^ This causes a series of rapid conformational changes that lead to the protein acquiring a catalytically active form which initially promotes autophosphorylation and homodimerization events.^11^ Subsequently, FLT3 exposes binding sites and phosphorylates signal transduction effector molecules involved in multiple pathways such as PI3K/AKT, MAPK/ERK, JAK/STAT, and PLC, among others (Figure 1B).^12^

It should be noted that, given its importance, FLT3 signaling is highly regulated by different mechanisms including a narrow range of FLT3‐expressing tissues and cells, the need for conformational changes for its activation, and the rapid internalization and degradation of the FLT3‐FL complex, among others.^9^, ^11^, ^13^ Despite all this, there are mutations that make FLT3 constitutively active and contribute to the development of diseases such as AML. Interestingly, although mutations in FLT3 may be driver mutations in AML, it is known that they cannot induce disease development on their own and require other helper mutations during leukemogenesis, including chromosomal alterations such as t(8; 21) (q22; q22); *RUNX1‐RUNX1T1*, inv(16) (p13.1q22); *CBFB‐MYH11* and t(15; 17) (q22; q12); *PML‐RARA*, as well as point mutations in genes such as *NPM1* and *DNMT3A* with which frequent coexistence has been found in patients with AML with normal karyotypes.^14^ The most common form of mutation affecting FLT3 is an internal tandem duplication (ITD) in exons 14 and 15 that affects the juxtamembrane region, while the second common type of mutation is a missense point mutation in exon 20 known as FLT3‐TKD because affects the second kinase domain.^15^, ^16^ Several studies have shown that FLT3‐ITD mutations have a greater clinical impact than FLT3‐TKD mutations, being associated with a poor clinical prognosis.^16^, ^17^ FLT3‐ITD can activate the same pathways as wild‐type FLT3, albeit in an aberrant manner. Also, because it is retained longer in the endoplasmic reticulum (ER)‐Golgi network, it can interact with other proteins and also activate other pathways.^18^ As a product of this, FLT3‐ITD acts to initiate a cycle of genomic instability through increased production of reactive oxygen species (ROS) and inefficient DNA damage repair^19^, ^20^ which, in turn, creates an environment conducive to the appearance of new mutations and clonal evolution.^21^

In this way, the purpose of this review is to discuss how FLT3‐ITD favors the generation of DNA damage and regulates repair mechanisms in such a way that the appearance of new clones that are more resistant to treatment and more aggressive is facilitated. Likewise, this article analyzes the feasibility of therapies that combine inhibitors of FLT3 signaling and inhibitors of DNA repair pathways.

## 2 FLT3‐ITD PROMOTES DNA DAMAGE

**FIGURE 2:**
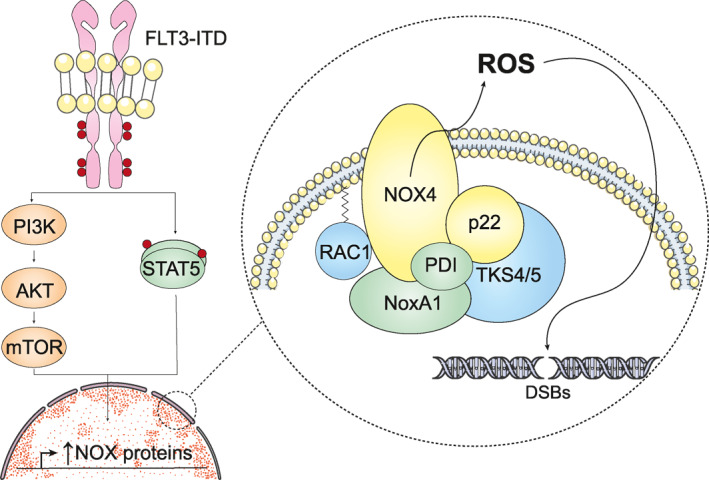
FLT3‐ITD promotes the formation of ROS. FLT3‐ITD, by activating PI3K/AKT and STAT5 signaling, promotes the transcription of NOX proteins, one of the main producers of ROS. In particular, NOX4D bound to the nuclear membrane produces ROS that help leukemia cells survive and damage DNA, mainly by producing double‐strand breaks

Every day, DNA integrity and stability are challenged by exogenous physical, chemical, or biological agents, as well as endogenous processes, including DNA replication errors, spontaneous hydrolytic reactions, and reactive oxygen species (ROS).^22^ It has been previously reported that FLT3‐ITD can promote DNA damage by different mechanisms. For example, intracellular ROS levels are increased in FLT3‐ITD cells compared to wild‐type FLT3 cells, where NOX family NADPH oxidases in the ER and mitochondria are one of the main sources of ROS generation.^23^ ROS are normally present at low and constant levels, playing an important role in cell signaling and homeostasis, but at high concentrations, ROS react rapidly with proteins, lipids, carbohydrates, and nucleic acids, causing irreversible functional alterations or even its complete destruction.^24^ FLT3‐ITD, by overactivating the STAT5 and PI3K/AKT pathways, maintains and sometimes increases the expression of p22phox and NOX proteins, thus favoring the production of ROS that diffuse into the nucleus and cause DNA damage, especially double‐strand breaks and mismatches (Figure 2).^19^, ^20^, ^25^, ^26^ Furthermore, the presence of FLT3‐ITD at the plasma membrane is required to maintain NOX protein levels and prevent proteosomal degradation of p22phox triggered by GSK3‐β signaling.^23^, ^27^ Overall, NOX proteins associate with p22phox to co‐stabilize in the membrane, and then RAC1 binds and exchanges GDP for GTP to initiate the conversion of oxygen to superoxide.^28^ In this regard, it has also been reported that RAC1‐GTP binding to p‐STAT5 is increased in FLT3‐ITD cells and, consequently, a greater amount of RAC1‐GTP is recruited to NADPH oxidase complexes.^19^ In particular, the nuclear membrane‐bound NOX4D isoform is overexpressed in patients and cell lines expressing FLT3‐ITD but is almost absent in their wild‐type FLT3‐expressing counterparts. It stands out that this protein generates ROS that favor the survival of leukemia cells.^25^ Due to a feedback mechanism, a vicious circle is created between FLT3 signaling and ROS production. In this way, ROS create an oxidative environment that enhances wild‐type FLT3 and FLT3‐ITD signaling (presumably by oxidation of certain cysteines such as residue 790) which, in turn, leads to increased ROS production.^29^

On the other hand, FLT3‐ITD expressing cells have high expression of succinate‐CoA ligases and high mitochondrial electron transport chain (ETC) complex II activity. Thus, a high respiratory activity is induced with increased production of mitochondrial ROS associated with the Krebs cycle and oxidative phosphorylation.^30^ Although there is no related report, there is a possibility that FLT3‐ITD, by increasing global transcription, may also cause DNA damage. In this sense, FLT3‐ITD could increase the number of transcription‐replication conflicts, activate responses to replicative stress and cause an accumulation of *R* loops, among other things.^31^ All this demonstrates that cells that express FLT3‐ITD present greater DNA damage than those that do not present the mutation, this being mainly caused by ROS, although other mechanisms may be associated.

## 3 FLT3‐ITD REGULATES DNA REPAIR PATHWAYS

Due to the important consequences that DNA damage can cause, cells have developed sophisticated strategies to repair it as quickly and precisely as possible, which are collectively known as DNA repair mechanisms and include several pathways (Table 1) whose functions include detecting DNA damage, signaling its presence, and promoting its repair.^32^ Because cancer must initially acquire permanent genomic mutations, it is, by definition, a disease due to inadequate DNA repair. In this manner, many cancers may be constantly acquiring new mutations.^33^ For this reason, it is interesting that the Cancer Genome Atlas (TCGA) reported that AML cells have a low average mutation frequency (approximately 1 mutation per megabase), these being mainly *C* > *T* transitions and affecting genes such as *PIK3CA*, *IDH1*, *IDH2*, *NPM1*, *FLT3*, *RUNX1*, *MIR142*, *DNMT3A*, *TET2*, *GATA3* and *MAP3K1* where on average two non‐synonymous mutations occur.^34^ This would indicate that DNA repair mechanisms function relatively well in AML, although several reports indicate that mutability is increased when FLT3‐ITD is expressed.^35^ FLT3‐ITD has been reported to increase interchromosomal homologous recombination events and these were also positively correlated with intracellular ROS levels.^36^ Similarly, patients with FLT3‐ITD positive AML have been reported to have a high frequency of rare structural chromosomal abnormalities at relapse.^37^ It appears that FLT3‐ITD cells have some repair pathways that work more efficiently than wild‐type FLT3 cells, while there are other pathways that do the opposite, thus it is important to identify which one can be used at what time and with what fidelity.^38^, ^39^ Another thing to keep in mind is that some DNA repair proteins, primarily involved in double‐strand break repair (both by homologous recombination [HR] and non‐homologous end joining [NHEJ]), mismatch repair (MMR), and base excision repair (BER) pathways, are susceptible to oxidative changes that regulate their activity. To mention a few, these are ATM, XRCC3, DNA‐PKcs, KU proteins, MGMT, AAG, OGG1, APE1, PARP1, and XRCC1.^40^

**TABLE 1:** Major DNA repair pathways in humans

Damage	Pathway	Subclassification	Type of damage it repairs	Main proteins involved
Single‐strand DNA breaks	Base excision repair (BER)	‐Short‐patch ‐ Long‐patch	Removal of a small base lesion that does not distort the helix	APEX1
				APEX2
				APTX
				FEN1
				HUS1
				LIG1
				LIG3
				MBD4
				MPG
				MUTYH
				NEIL1
				NEIL2
				NEIL3
				NTH
				OGG1
				PARP1
				PARP2
				PCNA
				PNKP
				POLB
				POLD1
				POLE
				POLH
				POLL
				RECQL2 (WRN)
				SMUG1
				TDG
				UNG
				XRCC1
	Nucleotide excision repair (NER)	‐ Global genome ‐ Transcription‐coupled	Removal of bulky DNA lesions that distort the helix	CSA
				CSB
				CUL4A
				DDB1
				DDB2
				ERCC1
				GTF2H1
				GTF2H2
				GTF2H3
				GTF2H4
				GTF2H5
				LIG1
				MMS19
				PCNA
				POLD1
				POLE
				RAD23 B
				RFC1
				RPA1
				XPA
				XPB
				XPC
				XPD
				XPF
				XPG
	Mismatch repair (MMR)	‐	Corrects DNA mismatches	EXO1
				LIG1
				MLH1
				MLH3
				MSH2
				MSH3
				MSH6
				PMS1
				PMS2
				POLD1
				POLE
				PCNA
				RPA
Double‐strand DNA breaks	Homologous recombination (HR)	‐‐‐	DSB repair using long regions of homology (precise mechanism). It can only be used during and shortly after DNA replication, during the S and G2 phases of the cell cycle.	BLM
				BRCA1
				BRCA2
				C19ORF40
				EME1
				EME2
				EXO1
				FANCA
				FANCB
				FANCC
				FANCD2
				FANCE
				FANCF
				FANCG
				FANCI
				FANCL
				MRE11 A
				MSH4
				MSH5
				MUS81
				NBS1
				PALB2
				RAD50
				RAD51
				RAD52
				RECQL
				RECQ4
				RECQ5
				RPA
				XRCC3
				XRCC2
				WRN
	Non‐homologous end joining (NHEJ)	‐ c‐NHEJ ‐ Alt‐NHEJ	DSB repair by microhomology (error prone) and operates throughout the cell cycle.	BRCA1
				BRCA2
				DCLRE1C (Artemis)
				DNA‐PKcs
				KU70 (XRCC6)
				KU80 (XRCC5)
				LIG4
				NHEJ1
				POLL
				POLM
				PRKDC
				RECQL2 (WRN)
				XPF
				XRCC4

**FIGURE 3:**
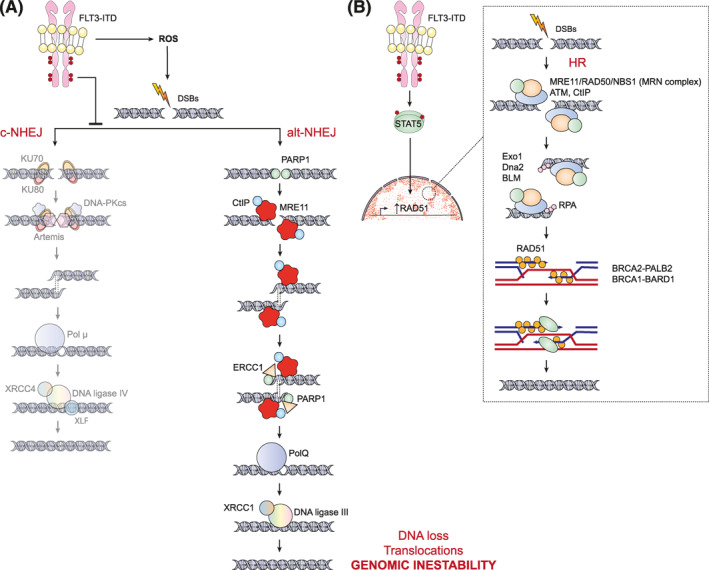
FLT3‐ITD causes defective DNA repair. Repair of double‐strand breaks in cells expressing FLT3‐ITD has higher errors because these damages are mainly repaired by alt‐NHEJ as a consequence of low levels of KU proteins (A). Meanwhile, HR repair is enhanced due to exacerbation of STAT5 signaling that contributes to RAD51 upregulation (B). HR can only act during and shortly after DNA replication, while alt‐NHEJ is any phase of the cycle

FLT3‐ITD upregulates the activity of the MMR pathway through the MAPK/ERK pathway that activates the transcription factor AP‐1 and its target genes, such as *MSH2*.^26^, ^41^ Meanwhile, cells expressing FLT3‐ITD have been reported to have greater errors in double‐strand break repair because these damages are primarily repaired by the alternative NHEJ (alt‐NHEJ) pathway as a consequence of low levels of KU proteins (Figure 3A). Notably, the resulting alterations are mainly deletions in regions of sequence microhomology.^39^ It should be noted that, like the classical NHEJ (c‐NHEJ) pathway, alt‐NHEJ can act at any phase of the cell cycle and in areas with high chromatin condensation.^42^ In contrast, HR repair occurs more efficiently in FLT3‐ITD cells than in wild‐type FLT3 cells (Figure 3B). Remarkably, the HR pathway can only be used during mitosis when chromosomes can line up and in areas without high chromatin condensation.^42^ One of the main reasons the HR pathway is enhanced is due to the exacerbation of STAT5 signaling caused by FLT3‐ITD. FLT3‐ITD can activate STAT5 either directly or indirectly (e.g., with the DOCK2/RAC1 axis), and through mechanisms other than wild‐type FLT3, contributes to the upregulation of RAD51.^26^ ^,^ ^38^ ^,^ ^43^ STAT5 signaling also activates CHK1, WEE1, PIM1, and RAD51, while its downregulation inhibits the expression of genes such as *BRCA1/2*, *RAD51*, and *BARD1* of the HR pathway, as well as *XRCC5* and *XRCC6* of the c‐NHEJ pathway.^44^, ^45^, ^46^ Furthermore, the high levels of PARP1 generated in FLT3‐ITD cells in response to extensive DNA damage cause STAT5 to be PARylated and this, at least in part, allows STAT5 signaling to remain persistently activated.^47^ Associated with these observations, in BaF3 pre‐B cells, the expression of FLT3‐ITD increased the levels of BRCA1 and PALB2 of the HR pathway, but reduced those of DNA‐PKcs, KU70 and LIG4 of the c‐NHEJ pathway.^48^ In patients with cytogenetically normal acute myeloid leukemia, in whom FLT3‐ITD is a common mutation, overexpression of genes from the BER, NER, Fanconi anemia (FA), MMR, and HR pathways was associated with poor clinical prognosis. In addition, the subclassification of these patients according to the presence or absence of NPM1 mutations and/or FLT3‐ITD revealed that the expression status of DNA repair genes can also influence the clinical prognosis of these groups, being better in patients with NPM1 mutations and low expression of DNA repair genes, while worse in patients with FLT3‐ITD and high expression of DNA repair genes.^49^ This may indicate that FLT3‐ITD cells with high DNA repair are better able to repair the extensive DNA damage constantly caused by ROS, conferring proliferation and survival advantages. Moreover, damage‐repair cycles coupled with defective repair in FLT3‐ITD cells could lead to the appearance of cell clones that are more resistant to treatment and more aggressive.

## 4 FLT3‐ITD FACILITATES CLONAL EVOLUTION

**FIGURE 4:**
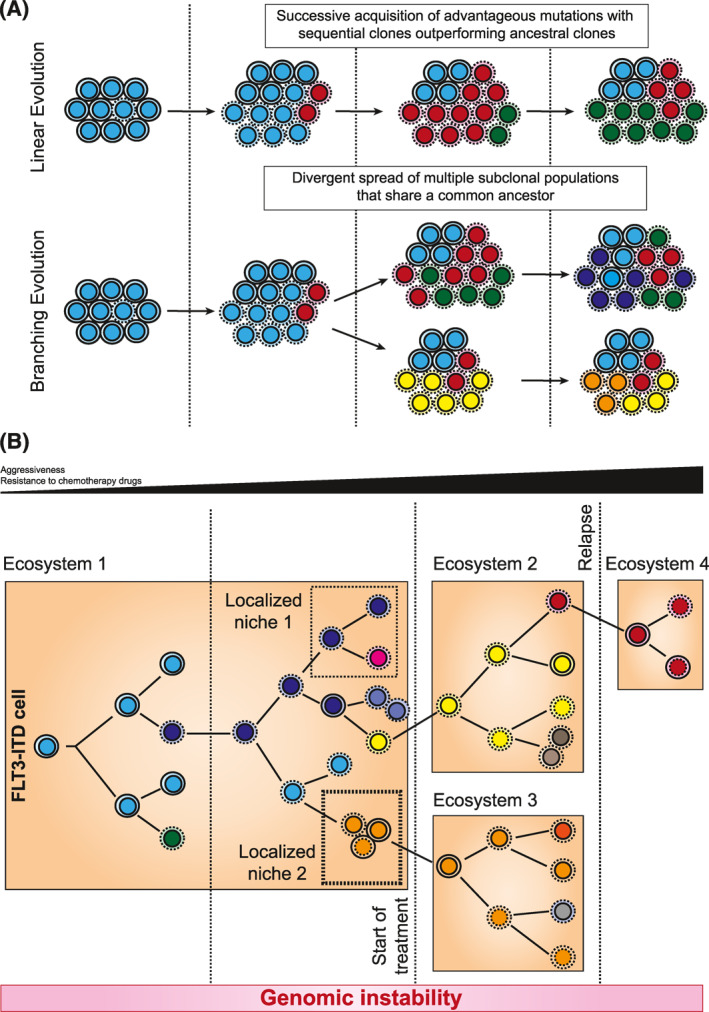
Clonal evolution of FLT3‐ITD cells. (A), Representation of linear and branched evolution models. (B), In FLT3‐ITD cells, selective pressures allow some mutant subclones to expand while others become extinct or inactive. Each different colored cell represents a genetically distinct subclone with different molecular signatures. The initiation of treatment exerts a great selective pressure that favors clonal expansion and genetic diversification. Subclones at relapse may originate at different times in the sequence from minor or major subclones of the primary cell. The vertical lines represent restrictions or selective pressures. There tends to be an increase in aggressiveness and drug resistance as clonal evolution proceeds

Cancers evolve through an iterative process of clonal expansion, genetic diversification, and clonal selection that results in a predominance of only those clones that are best adapted to the conditions of the surrounding microenvironment (constantly changing).^50^ There are two main models of clonal evolution, linear and branching (Figure 4A). The linear model involves the gradual acquisition of individual mutations, with the cell at the final step carrying all the mutations that arose during evolutionary history and outcompeting earlier clones that carry only some of the mutations.^51^ For its part, the branching model implies the eradication of the dominant clone, followed by the growth of a subclone.^52^ With little evolutionary pressure, it is likely to follow a linear evolution, but if the pressure changes profoundly (e.g., initiation of chemotherapy, stromal modification, major changes in growth factors, cytokines, or hormones), it is more likely that follow a branching evolution.^53^

Explaining this in more detail and in the context of FLT3‐ITD, the genetic instability that is generated by constant DNA damage and defects in its repair allows the appearance of a diverse collection of cells that harbor different molecular signatures (linear evolution) which then be selected in the surrounding microenvironment (branching evolution) (Figure 4B).^52^, ^54^ Thus, a clonal evolution can be clearly observed in patients studied at the time of diagnosis, during treatment and when they suffer relapses.^35^, ^55^ For example, one study mentioned that in patients without FLT3‐ITD more than half of the mutations were maintained from diagnosis to relapse, but in patients with FLT3‐ITD this did not occur and some mutations disappeared while others appeared.^55^ This would suggest that FLT3‐ITD promotes branching evolution. On the other hand, in FLT3‐ITD patients treated with midostaurin, a FLT3 inhibitor, the number of mutations that appeared at relapse was lower than in patients who did not receive the drug.^35^, ^55^ Furthermore, FLT3‐ITD persists in a considerable proportion of patients who relapse, which reinforces the conception of this as a driver mutation, although the number of ITD clones in the same patient decreases.^55^ This would suggest that linear evolution also occurs in FLT3‐ITD patients and thus indicate that, whether linear or branched, cell populations are constantly undergoing clonal evolution to better adapt to their environment.

Increased homologous interchromosomal recombination enables loss of heterozygosity (LOH) during relapse by deleting the allele encoding wild‐type FLT3, and this is associated with a more aggressive phenotype.^36^ Remarkably, the selection of these clones is accelerated by the administration of chemotherapeutic drugs, providing a powerful source of artificial selection, and resulting in a selective pressure that allows only variant cells that resist treatment to proliferate and cause massive death of cells that do not do it.^50^, ^56^, ^57^ In this sense, the appearance of clones that present additional mutations in FLT3 and/or in other genes makes FLT3 inhibitors and other chemotherapeutic drugs stop working in patients. These additional mutations (in addition to FLT3‐ITD) include FLT3‐TKD mutations such as N676K, which reduce the efficacy of midostaurin, F621L, A627P, F691L, and Y842C, which confer resistance to midostaurin, sunitinib, sorafenib, lestaurtinib, quizartinib, KW2449, and AGS324, as well as G697R and D835, which have been identified as a mutation that confers high‐level resistance to several FLT3 inhibitors.^58^, ^59^, ^60^, ^61^, ^62^, ^63^ Patients with FLT3‐ITD have been reported to have a higher frequency of cytogenetic evolution and progression to adverse karyotype during relapse compared with those lacking this mutation.^64^, ^65^ Furthermore, FLT3‐ITD‐positive AML is characterized not only by a high relapse rate and short relapse‐free survival, but also by a lower probability of responding to treatment after relapse, resulting in poorer survival.^64^ A study comparing the genetic background of FLT3‐ITD patients at disease onset and after relapse reported an increase in *C* > *T* transitions along with *C* > *A* transversions and the appearance of clones with deleterious mutations in tumor suppressor genes such as *WT1*, *ASXL1*, *SETD1A*, *MLL3*, *JHDM1D*, *EPHB1*, *KDM6A*, *FAT1*, and *XPO1*.^35^

## 5 THERAPY WITH FLT3 INHIBITORS AND DNA REPAIR INHIBITORS

Conventional induction therapy for AML consists of a dose of 100–200 mg/m^2^ of the arabinoside cytarabine in continuous infusion for 7 days, accompanied by one of the following two anthracyclines for 3 days, idarubicin at 12 mg/m^2^ or daunorubicin at doses of 45–60 mg/m^2^, a therapy commonly called 7 + 3.^66^ In the case of newly diagnosed patients with FLT3‐ITD, the administration of the FLT3 inhibitor midostaurin has been approved together with conventional induction therapy, as well as gilteritinib, which is a more potent and specific FLT3 inhibitor, as monotherapy for relapsed/refractory FLT3‐ITD positive AML.^67^ In this sense, inhibitors of FLT3 have been shown to partially reduce the number of errors caused by the alt‐NHEJ pathway, but cells resistant to them exhibit increased activation of this pathway.^39^, ^47^ Similarly, midostaurin inhibited the HR pathway by reducing RAD51 expression and activity, reversing the daunorubicin‐resistant phenotype.^38^ Inhibitors of FLT3 also reduced SIRT1 levels and favored its association with ATM‐phosphorylated DBC1 to maintain p53 residue K382 acetylated and promote genotoxic stress‐induced apoptosis.^68^

Although these protocols have improved outcomes for patients with FLT3‐ITD, different options combining different drugs and/or therapies to increase survival rates are still being explored. Based on this, the inhibition of the DNA repair machinery is an option that has been considered since it would collaborate with chemotherapy drugs and would allow the DNA damage to be so great (due to the action of FLT3‐ITD itself, the cell metabolism and chemotherapy drugs) that it would lead to senescence and apoptosis of leukemic cells. One of the targets being explored for this type of therapy is PARP1, a nuclear protein that functions through poly‐ADP‐ribosylation (PAR) of itself and other proteins to catalyze single‐ and double‐strand break repair.^69^ In BaF3 cells, the use of quizartinib, a selective FLT3 inhibitor, reduced the levels and activity of several HR and NHEJ proteins (BRCA1, BRCA2, PALB2 and RAD51, KU70, LIG4), making them more susceptible to action of PARP1 inhibitors, particularly on proliferating and quiescent leukemia stem cells. Notably, this did not occur in cells expressing wild‐type FLT3.^48^ Meanwhile, the FLT3 inhibitor AIU2008 also downregulated the HR and NHEJ pathways, synergizing with PARP inhibitors to inhibit leukemic cell growth.^70^ Other compounds with FLT3 inhibitory activity showed synthetic lethality with PARP inhibitors involving the aforementioned DNA repair pathways, inhibition of STAT5 signaling, and YAP1 deacetylation.^71^, ^72^ On the other hand, use of a DNA‐PKcs inhibitor such as M3814 was shown to enhance DNA damage signaling, synergize the effects of topoisomerase II inhibitors and sensitize AML cells to P53‐dependent apoptosis, regardless of FLT3 mutational status, the surrounding cellular context, and its combination with cytarabine.^73^ The same was true for the combination of M3814 and the DNA‐intercalating agent calicheamicin.^74^ Unfortunately, almost all these results of combined treatments between FLT3 inhibitors and inhibitors of the DNA repair machinery have been in cell lines and a few in mice, so many more experiments are still required to elucidate whether combined therapy is a viable proposal.

## 6 CONCLUSIONS AND FUTURE PERSPECTIVE

Improving the survival of AML patients represents a major challenge that many researchers around the world are working hard on. In this sense, FLT3‐ITD is a relevant mutation that is associated with a poor clinical prognosis and, despite all the knowledge that has been generated around it, there are still many areas to explore. It should be noted that the World Health Organization (WHO), although it did not designate a special category for patients with the FLT3‐ITD mutation, mentions it extensively in the AML classification.^75^ Current guidelines recommend FLT3‐ITD testing at diagnosis and earlier incorporation of specific agents to achieve deeper remissions and early consideration for allogeneic stem cell transplantation (ASCT). Furthermore, FLT3‐ITD promotes further DNA damage, primarily through ROS overproduction, and at the same time downregulates some of the DNA repair pathways leading to defective repair. All this generates an environment conducive to the creation of a large number of clones with heterogeneous molecular characteristics that, under the environment of great evolutionary pressure due to chemotherapeutic drugs, allows a rapid clonal evolution that is associated with a poor clinical prognosis.

One of the areas to investigate and that would provide important information, which is why it is discussed in this review, is clonal evolution. Clonal evolution is complicated and complex to study, which is reflected in the few studies that currently exist in this area. In this context, although the different investigations can be oriented in many directions, we will highlight only three here:

The first category would be to analyze how clones evolve and which mechanism of evolution is preferred (linear or branched), for which it would be necessary to identify the different molecular signatures and how they behave at different points of the disease, such as diagnosis, at various points in treatment and during relapse. Also, it would be interesting to know the role of other types of mutations in FLT3 with respect to DNA damage and its repair and to compare them with those of FLT3‐ITD. To do this, tools such as single‐cell sequencing would be of great help, along with artificial intelligence and machine learning systems that can extrapolate these results to other patients and make predictions to provide more personalized treatment.

The second category is to elucidate the mechanisms by which clones are selected, which could mainly consider the context of the cellular microenvironment in the bone marrow, as well as the effect of chemotherapeutic drugs and/or radiation. To this end, three‐dimensional cell culture systems in bone marrow tissue, gene editing (CRISPR‐Cas9, TALENs, ZFNs) and high‐throughput animal models could provide important insights. Previous RNA‐seq, ChIP‐seq, microarray, epigenomic, proteomic and metabolomic data in this context could also be collected, homogenized and analyzed together.

The third category involves, based on clonal evolution, selecting the appropriate combinations of drugs and/or therapies. Although AML therapy protocols are well established (7 + 3 therapy), it would be good to know if they can be customized (e.g., by adding combined therapies with FLT3 inhibitors and inhibitors of some DNA repair pathways). Identifying whether it is possible to control clonal evolution with drugs would also be an interesting option.

Clearly, all of these categories take time and a lot of work and effort, but as we learn more about the evolution of AML disease, more patients will have better survival and disease‐free survival.

## AUTHOR CONTRIBUTIONS

Francisco Alejandro Lagunas‐Rangel conceptualized the idea, looked up the references and wrote the article.

## CONFLICT OF INTEREST

The author declares no conflict of interest.

### PEER REVIEW

The peer review history for this article is available at https://publons.com/publon/10.1002/hon.3076.

## ETHICS STATEMENT

This is a review manuscript based on previously published articles so ethical approval is not required.

## Data Availability

Data sharing not applicable to this article as no datasets were generated or analyzed during the current study.
